# Analysis of co-assembly and co-localization of ameloblastin and amelogenin

**DOI:** 10.3389/fphys.2014.00274

**Published:** 2014-07-25

**Authors:** Parichita Mazumder, Saumya Prajapati, Sowmya Bekshe Lokappa, Victoria Gallon, Janet Moradian-Oldak

**Affiliations:** Division of Biomedical Sciences, Center for Craniofacial Molecular Biology, Herman Ostrow School of Dentistry, University of Southern CaliforniaLos Angeles, CA, USA

**Keywords:** ameloblastin, amelogenin, protein-protein co-assembly, confocal microscopy, quantitative co-localization analysis

## Abstract

Epithelially-derived ameloblasts secrete extracellular matrix proteins including amelogenin, enamelin, and ameloblastin. Complex intermolecular interactions among these proteins are believed to be important in controlling enamel formation. Here we provide *in vitro* and *in vivo* evidence of co-assembly and co-localization of ameloblastin with amelogenin using both biophysical and immunohistochemical methods. We performed co-localization studies using immunofluorescence confocal microscopy with paraffin-embedded tissue sections from mandibular molars of mice at 1, 5, and 8 days of age. Commercially-available ameloblastin antibody (M300) against mouse ameloblastin residues 107–407 and an antibody against full-length recombinant mouse (rM179) amelogenin were used. Ameloblastin-M300 clearly reacted along the secretory face of ameloblasts from days 1–8. Quantitative co-localization was analyzed (QCA) in several configurations by choosing appropriate regions of interest (ROIs). Analysis of ROIs along the secretory face of ameloblasts revealed that at day 1, very high percentages of both the ameloblastin and amelogenin co-localized. At day 8 along the ameloblast cells the percentage of co-localization remained high for the ameloblastin whereas co-localization percentage was reduced for amelogenin. Analysis of the entire thickness on day 8 revealed no significant co-localization of amelogenin and ameloblastin. With the progress of amelogenesis and ameloblastin degradation, there was a segregation of ameloblastin and co-localization with the C-terminal region decreased. CD spectra indicated that structural changes in ameloblastin occurred upon addition of amelogenin. Our data suggest that amelogenin-ameloblastin complexes may be the functional entities at the early stage of enamel mineralization.

## Introduction

Tooth enamel, the hardest substance in the body, is formed by an evolutionarily highly conserved biomineralization process that is controlled by extracellular matrix proteins. During the secretory stage of enamel formation, the three major proteins secreted by ameloblasts are amelogenin (Amel) (Snead et al., 1985), ameloblastin (Ambn) (Krebsbach et al., 1996), and enamelin (Hu et al., 2001a,b; Hu and Yamakoshi, 2003). Proper mineralization of enamel depends upon the secretion of these proteins, as well as their being processed into smaller functional components and eventually degraded by proteinases such as enamelysin (matrix metalloproteinase 20, Mmp-20) and kallikrein 4 (Klk4) (Bartlett and Simmer, 1999). It was suggested that those proteins of enamel must have specific protein–protein interactions to assemble an organic matrix that is capable of undergoing mineral replacement and forming the highly ordered three-dimensional structure of the hydroxyapatite crystallites (Hu et al., 2001a; Bouropoulos and Moradian-Oldak, 2004; Bartlett et al., 2006; Fan et al., 2008; Iijima et al., 2010; Yang et al., 2011; Gallon et al., 2013). However, there is still a gap in our knowledge as how these enamel matrix components interact with one another to form an assembled matrix that initiate and orchestrate the events of mineralization (Paine et al., 2001; Yamakoshi et al., 2003; Ravindranath et al., 2004). We have recently reported cooperative function of amelogenin and enamelin as well as their interactions using both *in vitro* and *in vivo* strategies (Bouropoulos and Moradian-Oldak, 2004; Fan et al., 2011; Yang et al., 2011; Gallon et al., 2013).

Ameloblastin is a member of the secretory calcium-binding phosphoprotein (SCPP) family of proteins (Kawasaki and Weiss, 2003). It is a typical extracellular matrix (ECM) protein that may be involved in the regulation of adhesion, proliferation, and differentiation of ameloblasts (Fukumoto et al., 2004), and it seems to serve essential developmental functions of enamel. Support for this notion was provided by the finding that an enamel layer fails to appear on the teeth of mice that are genetically engineered to produce a truncated form of ameloblastin (exon 5 and 6 deleted) (Smith et al., 2009; Wazen et al., 2009). Inactivation of the ameloblastin gene leads not only to loss of production of the full-length protein by ameloblasts but also to a reduction in the expression levels of amelogenin with no apparent change in the levels of other proteins (Fukumoto et al., 2004; Zalzal et al., 2008).

A potential mechanisms by which ameloblastin functions as an ECM protein in tooth enamel has been identified, including involvement in mineralization by means of calcium-binding sites at the C-terminus of ameloblastin (Yamakoshi et al., 2001; Hu et al., 2005; Kobayashi et al., 2007; Tamburstuen et al., 2010). Ameloblastin is rapidly processed after secretion. Full-length ameloblastin is only found adjacent to the non-secretary face of the Tomes' processes of the ameloblasts (Uchida et al., 1995; Hu et al., 1997; Murakami et al., 1997), while lower molecular weight proteins are present in the sheath space and in the rods of the superficial layer. The porcine N-terminal cleavage products (13, 15, 17 kDa) are stable and concentrate in the prism sheath. In contrast, the C-terminal cleavage products (40, 50 kDa) are successively cleaved into smaller peptides (8, 13, 15, 27, 29 kDa) and lost from the immature enamel soon after secretion (Uchida et al., 1998).

Our present study focuses on the co-localization of amelogenin and ameloblastin and their interactions. We propose that such interactions are important for the formation of highly organized enamel mineral and for maintaining its prismatic structure. We hypothesized that, by analyzing the spatial co-relation between ameloblastin and amelogenin in the enamel matrix using a well-established *in vivo* approach, complemented by investigating the interaction between full-length ameloblastin and amelogenin using *in vitro* experiments, we will gain new and important information on the roles played by ameloblastin-amelogenin complexes in normal dental enamel formation. We used immunofluorescence confocal microscopy with mouse mandibular first molars at differing postnatal ages (P1, P5, and P8) and two antibodies to ascertain when both ameloblastin and amelogenin are secreted into the ECM of enamel, as well as whether they are co-localized, which would support the possibility of their interaction *in vivo*. Commercially available antibody against 107–407 residues of mouse ameloblastin and an antibody against the full-length recombinant mouse (rM179) amelogenin were used in this procedure (Simmer et al., 1994). Direct *in vitro* evidence of structural changes in ameloblastin was observed upon addition of amelogenin using far-UV circular dichroism (CD) spectra.

## Materials and methods

### Description of antibodies

**Figure 1:**
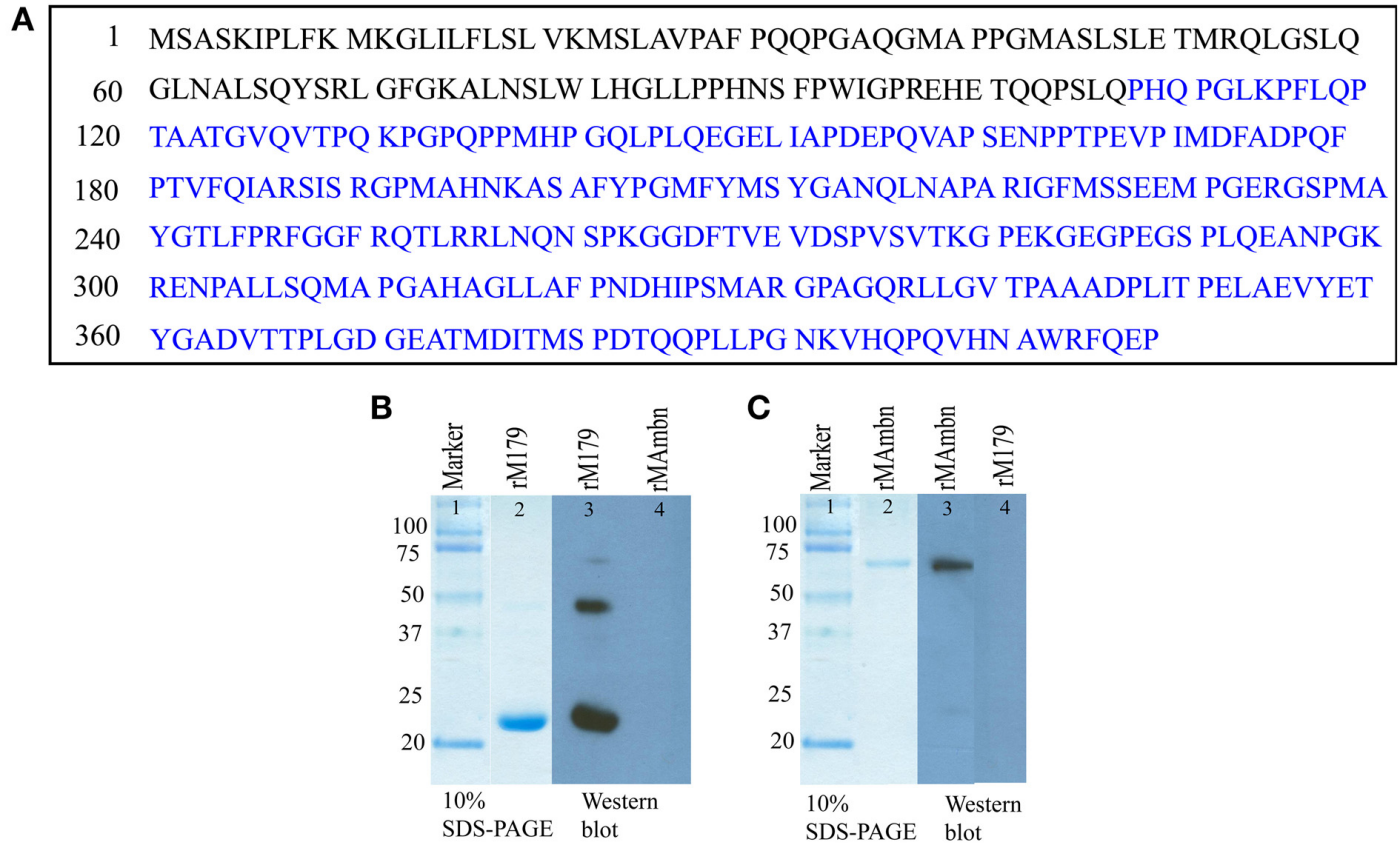
**(A)** Sequence of full-length mouse ameloblastin (Gene ID: 11698), the epitope of anti-ameloblastin antibody M300 is indicated by blue color. Western blot analysis with **(B)** anti-amelogenin antibody (lane 3, 4) and **(C)** M300 antibody (lane 3, 4) to check cross reactivity of the antibodies with recombinant proteins.

Two primary antibodies were used in this study, (i) rabbit anti-ameloblastin (M300), commercially available antibody against the portion of mouse ameloblastin extending from residues 107–407 (Figure 1A, residues labeled with blue) (Santa-Cruz Biotechnology, Inc. Santa Cruz, California) and (ii) chicken anti-amelogenin generated against full-length mouse amelogenin (a gift from Prof M. Snead). For immunohistochemical staining two secondary antibodies were used (i) goat anti-rabbit conjugated with Texas Red and (ii) bovine anti-chicken conjugated with FITC (fluorescein isothiocyanate) (Santa-Cruz Biotechnology, Inc. Santa Cruz, California).

We checked whether there was any cross-reaction between antibodies and the proteins, by using Western blot with recombinant mouse amelogenin and ameloblastin (Figures 1B,C, lane 4-5). Samples were electrophoresed on 10 % polyacrylamide gel containing 0.1% sodium dodecyl sulfate (SDS-PAGE) under non-reducing conditions and then electrotransferred to polyvinylidene fluoride (PVDF) membranes (PALL, Life Sciences, BioTrace™ PVD 0.45 μm) in semi-dry transblotter (Bio-Rad Scientific Instruments) at 10 V for 45 min. After blocking with phosphate-buffered saline, 0.1% Tween 20 (PBST) (pH 7.4) with 5% non-fat milk for 2 h at 37°C, the membrane was washed five times with PBST. After washing, the membranes were treated with the primary antibody (M300, rabbit anti-ameloblastin) against mouse ameloblastin at a 1:1000 dilution overnight at 4°C. After five washes, the membranes were incubated for 2 h in anti-chicken (for amelogenin) and anti-rabbit (for ameloblastin) secondary antibody conjugated with horseradish peroxidase at an appropriate dilution (1:3000). After another wash, the membranes were incubated in Amersham™ ECL Western blotting detection reagents (GE healthcare). In lane 4 from Figures 1B,C, there was no protein band, demonstrating that the both ani-amelogenin and M300 antibody did not react with recombinant ameloblastin and amelogenin respectively.

### Protein expression and purification

Preparation of the recombinant rM179 has been described elsewhere (Moradian-Oldak et al., 2000; Lakshminarayanan et al., 2010). The protein was further purified on a Jupiter C4 semi-preparative reversed phase column (10 × 250 mm, 5 μm) Varian Prostar HPLC system (ProStar/Dynamics 6, version 6.41, Varian, Palo Alto, CA). A linear gradient of 60% acetonitrile in 0.1% trifluoroacetic acid at a flow rate of 2 ml/min was used (Figure 1B, lane 2).

Recombinant porcine amelogenin (rP172) was expressed in *Escherichia coli* strain BL21-codon plus (DE3-RP, Agilent Technologies, Inc., Santa Clara, CA), precipitated by 20% ammonium sulfate and purified as previously described (Hu et al., 1996; Ryu et al., 1999). The precipitate was dissolved in 0.1% TFA and protein purification was accomplished on a reverse phase C4 column (10 × 250 mm, 5 μm) mounted on a Varian Prostar HPLC system (ProStar/Dynamics 6, version 6.41 Varian, Palo Alto, CA) and fractionated using a linear gradient of 60% acetonitrile at a flow rate of 1.5 mL/min.

Recombinant murine ameloblastin (rMAmbn) both phosphorylated and glycosylated (Figure 1C, lane 2) was prepared in a Drosophila melanogaster expression system using Schneider 2 cells (Invitrogen, CA) as previously described (Zeichner-David et al., 2006). The protein was purified by HPLC with a gradient of 20–80% B for 60 min (buffer B was 60% v/v aqueous acetonitrile in 0.1% v/v trifluoroacetic acid (TFA) and buffer A was 0.1% TFA) at a flow rate of 1.0 ml/min.

Sodium dodecyl sulfate–polyacrylamide gel electrophoresis (SDS–PAGE) was carried out in the presence of 0.1% SDS, using a 10% acrylamide gel (Laemmli, 1970) to characterize the proteins (Figures 1B,C). The gel was stained with Coomassie brilliant blue.

### Immunochemical analysis of protein from enamel extracellular matrix at day 9

**Figure 2:**
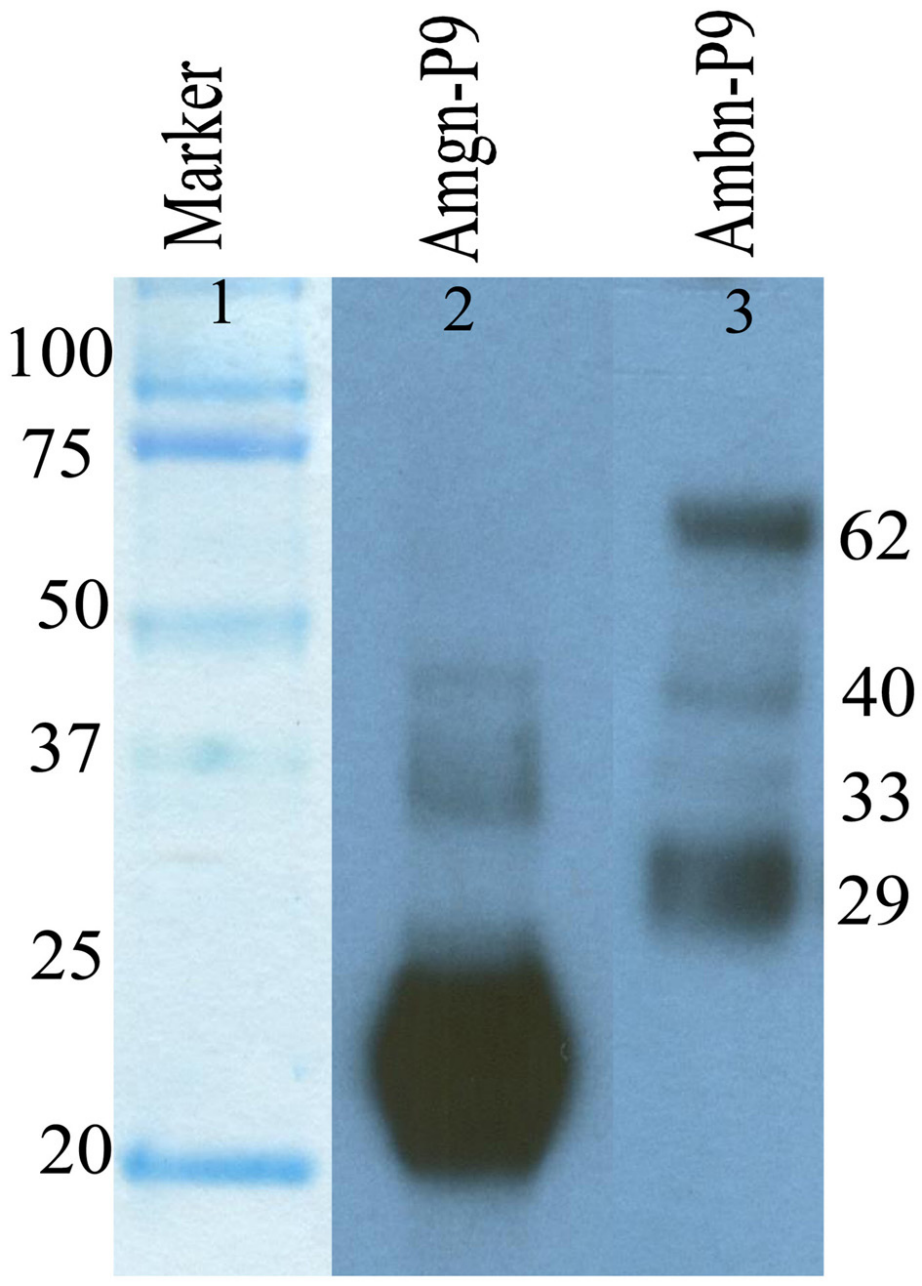
**Immunochemical detection of amelogenin (lane 2) and ameloblastin (lane 3) proteins and their degradation products present in enamel matrix from postnatal day 9 old (P9) mouse enamel**.

For immunochemical analyses, six 9-day-old (postnatal) mice were used. All mouse studies were conducted according to protocols approved by the USC Institutional Animal Care & Use Committee. The upper and lower molars were extracted from the alveolar bone. The enamel surface was then gently wiped to remove remnants of secretory ameloblasts. The sample was dissolved in 0.5 M acetic acid (four molar /100 μl) and dissolution is aided by grinding the samples in the acid with pestle and then total extracted protein was lyophilized. Samples were electrophoresed on 10 % polyacrylamide gel containing 0.1% sodium dodecyl sulfate (SDS-PAGE) under non-reducing conditions and then Western blot analysis was performed as described in previous Section using both anti-amelogenin and M300 primary antibody (Figure 2, lane 2, 3).

### Tissue preparation

Mandibular processes of mice from postnatal days 1–8 (here the day of birth was termed postnatal day 0) were dissected and fixed in 4% paraformaldehyde in PBS at pH 7.4 for 24 h. Samples from day 5–8 were demineralized in 0.2% paraformaldehyde, 0.05% glutaraldehyde and 10% EDTA for ~1–2 weeks (pH 8.0) at 4°C with rocking. The tissues were then processed for histological analysis and embedded in paraffin. Tissue sections of 7 μm thickness were cut from the wax blocks and mounted onto glass slides.

### Immunofluorescence labeling

Tissue sections were subjected to an antigen retrieval step by incubation in 10 mM sodium citrate with 0.05% Tween 20 at pH 6.0 in a 60°C water bath overnight. The sections were allowed to cool in dH_2_O before being rinsed in TBS and then incubated with 0.3% H_2_O_2_ for 15 min. After washing with TBS, sections were blocked with 1% bovine serum albumin (BSA) for 15 min before incubation overnight at room temperature with primary antibody. We have used 1:500 dilutions for anti-ameloblastin M300 and 1:1000 for anti-amelogenin (Section Description of Antibodies). For simultaneous immunofluorescence double-labeling mixture of both primary antibodies with above mentioned dilution were used. After another wash with TBS, sections were incubated with secondary antibody (for ameloblastin goat anti-rabbit-Texas Red and for amelogenin bovine anti-chicken-FITC with 1:100 dilution each) for 3 h at room temperature, before mounting with Vectashield® Hard Set™ mounting medium with DAPI. Similar to primary antibody we have used mixture of both secondary antibodies for immunofluorescence double-staining. Several controls were performed to ascertain the specificity of the antibodies, which included incubating each primary antibody with both secondary antibodies, as well as each secondary antibody with both primary antibodies. The sections were visualized using a Leica TCS SP5 confocal microscope.

### Quantification of co-localization of two antigens

Using the Leica Application Suite Advanced Fluorescence software (version 2.5.2.6939) confocal images were analyzed with a threshold of each channel at 30% and a background correction of 20%, and scatter grams were generated. Mander's colocalization coefficients were calculated to predict the contribution of each particular antigen to the areas with co-localization, using the following equations (Manders et al., 1993):

$$M_{red}=\frac{\sum_{i}R_{i,\;coloc}}{\sum_{i}R_{i}};M_{green}=\frac{\sum_{i}G_{i,\;coloc}}{\sum_{i}G_{i}}$$

when, *R*_*i*, *coloc*_ = *R*_*i*_ if *G*_*i*_ > 0; *G*_*i*, *coloc*_ = *G*_*i*_ if *R*_*i*_ > 0, i.e., *M*_*red*_ is the sum of intensities of red pixels that have a green component divided by the total sum of red intensities.

### Circular dichroism

CD measurements were carried out at 37°C on a JASCO J-715 spectropolarimeter between 190 and 260 nm in a quartz cell with a path length of 1 mm. Four spectra were accumulated for each sample, and the contribution of the buffer was always subtracted. Data are represented as mean residue ellipticities. Stock solution of rP172 (89% analogues to mouse amelogenin) was prepared in distilled water and rMAmbn was prepared in Tis-HCL pH 8.0. CD experiments were performed with 10 μM protein in 10 mM potassium phosphate buffer at pH7.4. CD spectra were normalized to the protein concentration and normalized spectra were deconvoluted using the CDSSTR algorithm and the Reference set 7 at Dichroweb (Lobley et al., 2002; Whitmore and Wallace, 2004). The values for helix1 and helix2 as well as sheet1 and sheet2 were added to obtain total helix and total sheet content (Sreerama et al., 1999).

## Results

### Degradation and distribution of mouse ameloblastin

We analyzed the degradation of mouse ameloblastin immunochemically using Western blot analysis (Figure 2, lane 3) with proteins extracted from 9 day old mouse enamel. In the enamel matrix sample, M300 antibody reacted with protein bands having molecular weights of near 62, 40, 33, and 29 kDa (Figure 2, lane 3). The largest 62 kDa band corresponds to the nascent ameloblastin and the others are presumably C-terminal ameloblastin processing products (Uchida et al., 1997, 1998; Brookes et al., 2001).

**Figure 3:**
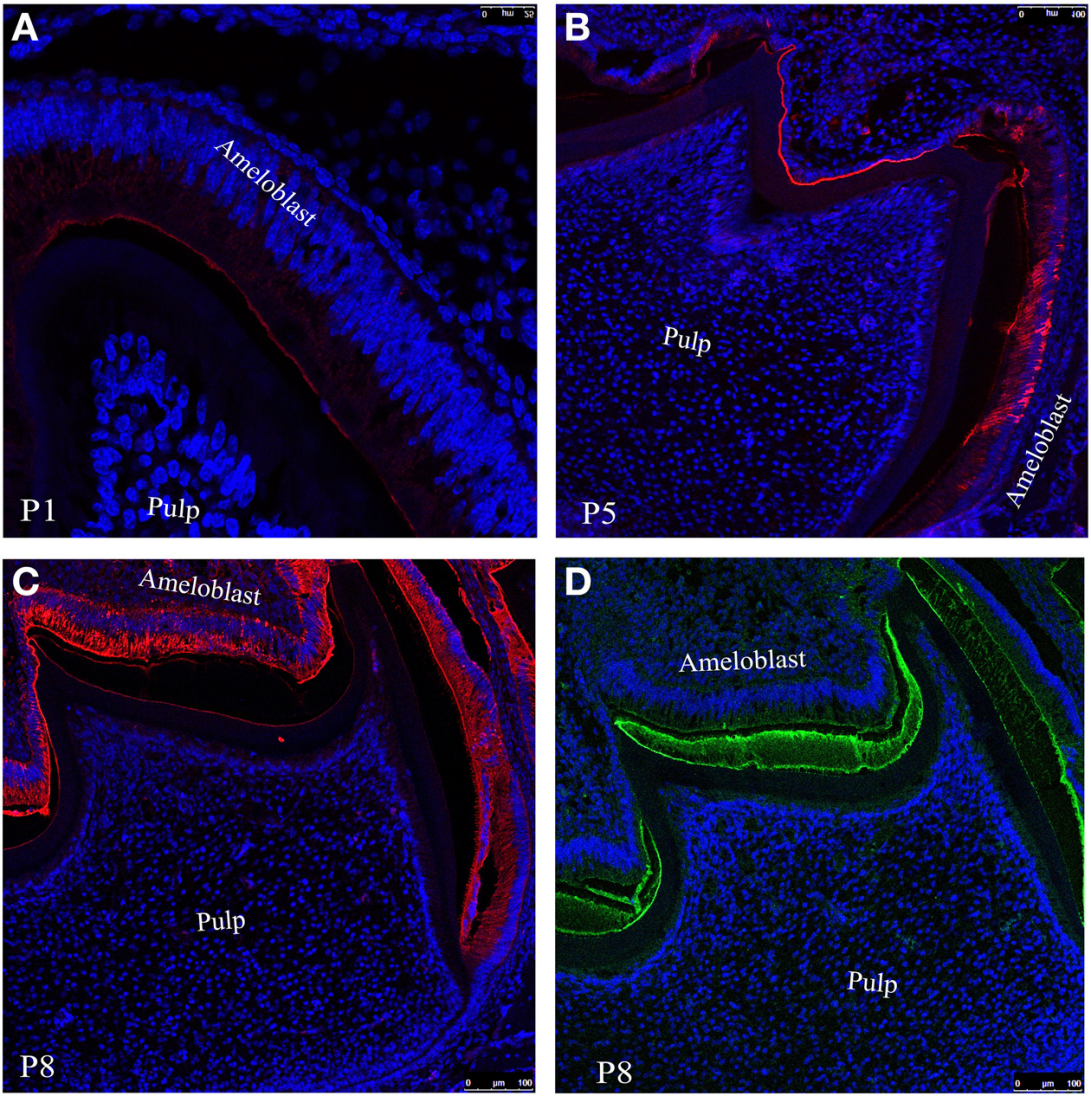
**Confocal images of mouse molar enamel showing immunofluorescence of ameloblastin at postnatal days (A) P1, (B) P5 and (C) P8**. **(D)** Represents the immunofluorescence of amelogenin at P8. Nuclei are stained with DAPI (blue).

To observe the distribution of ameloblastion and its proteolytic products in enamel we have incubate tissue sections from day 1 (Figure 3A), 5 (Figure 3B) and 8 (Figure 3C) with anti-ameloblastin M300 antibody followed by immunofluorescence labeling with Texas red conjugated anti-rabbit secondary antibody (see Section Description of Antibodies and Immunofluorescence Labeling). Confocal image of the section at postnatal day 1(P1) indicated that M300 antibody stained the distal cytoplasm of ameloblasts, particularly in Tomes' processes and immature enamel near the secretory surfaces of pre-secretoty ameloblasts (Figure 3A). During the early maturation stage when the degradation of ameloblstin has already started, at day 5 (P5), they were present in the enamel epithelium and at the enamel surface (Figure 3B). At day 8 (P8), we observed that M300 reacted with the proteins which were accumulated in the juxtanuclear cytoplasm, the distal cytoplasm near the enamel surface, at the enamel surface and dentino-enamel junction (Figure 3C) (Lee et al., 1996).

The immunofluoresence study at P8 (Figure 3C) with M300 antibody gives us a clear picture of localization pattern of ameloblastin and its C-terminal processing products which were identified by immunochemical analysis (Figure 2, lane 3). Figure 3D shows the localization pattern of amelogenin and its processing products (Figure 2, lane 2) at day P8. The anti-amelogenin antibody stained evenly across the enamel layer (visualized by green color FITC conjugated secondary antibody).

### The co-localization pattern of Ambn and Amgn in mouse mandibular molars

**Figure 4:**
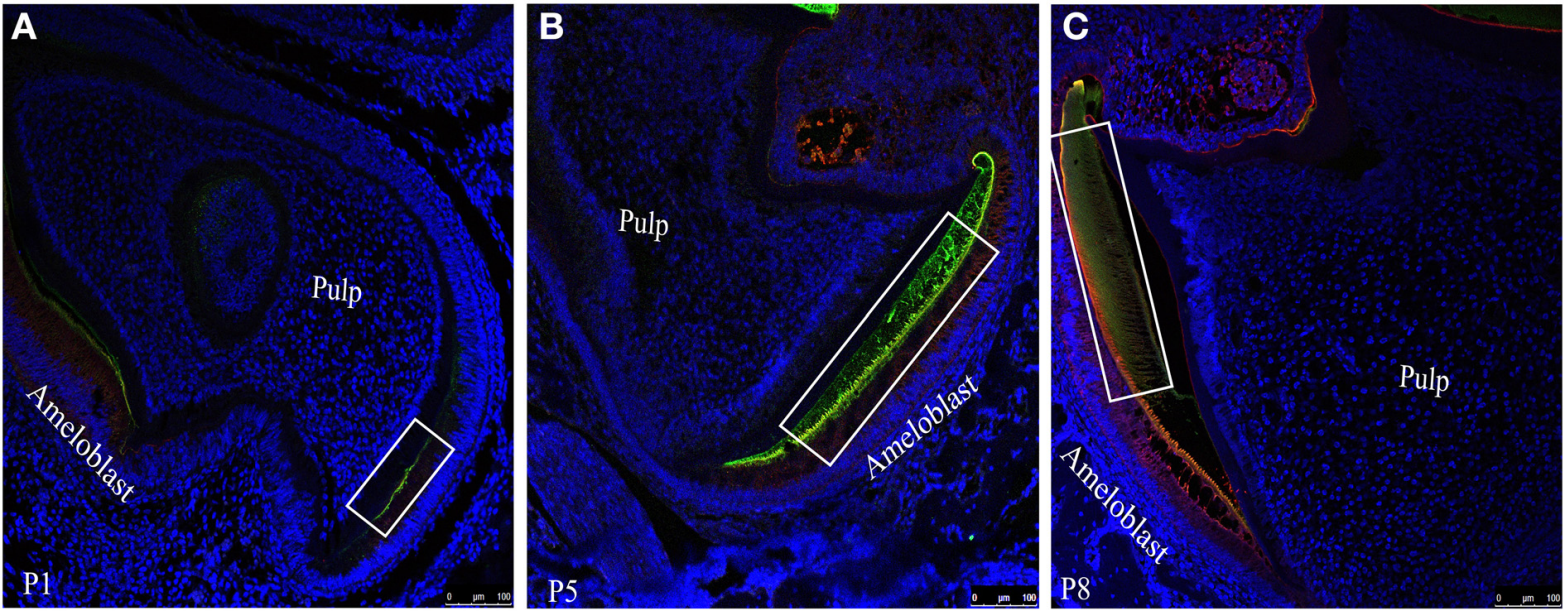
**Co-localization patterns (yellow color) of ameloblastin and amelogenin within the forming enamel layer of mouse mandibular molar sections at (A) P1, (B) P5 and (C) P8**. Amelogenin and ameloblstin simultaneously co-labeled with FITC (green) and with TR (red) respectively.

**Figure 5:**
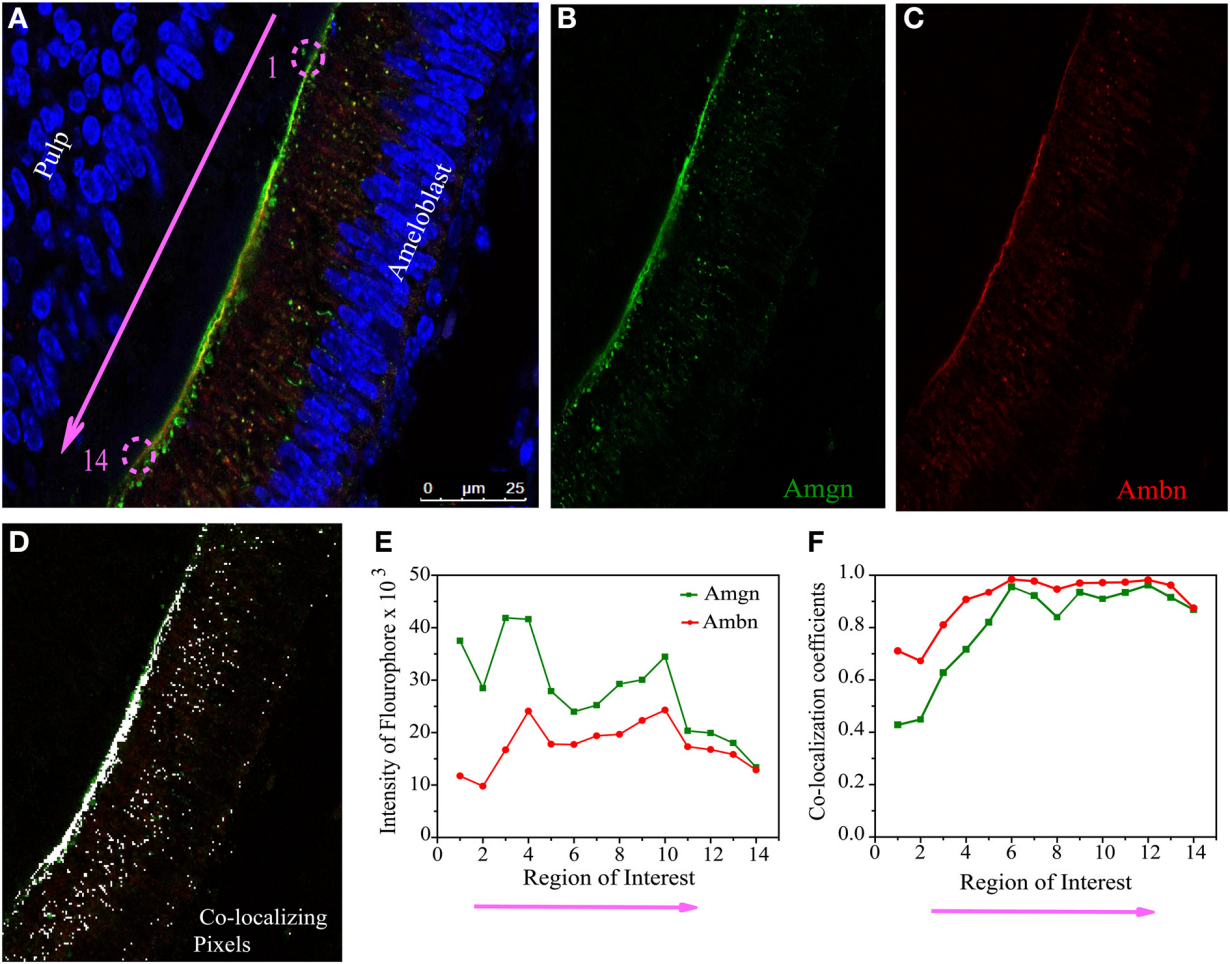
**Quantitative co-localization of ameloblastin and amelogenin in mouse molar enamel at P1. (A)** Confocal image with higher magnification taken from the sample in Figure 4A (see square), considered for QCA analysis along the enamel surface (pink arrow). The individual channels detecting **(B)** only Amgn and **(C)** only Ambn signal. **(D)** Represent the pixels that have only co-localizing signal after threshold and background corrections of image **(A)**. **(E)** Graph of total intensity sum of each fluophore. **(F)** Plot of Mander's co-localization coefficient with respect to region of interest as shown in **(D)**. Each ROI dimension (5 × 5 μm). Note that ROI 1 to 14 in **(E,F)** correspond to the pink arrow in **(A)**.

**Figure 6:**
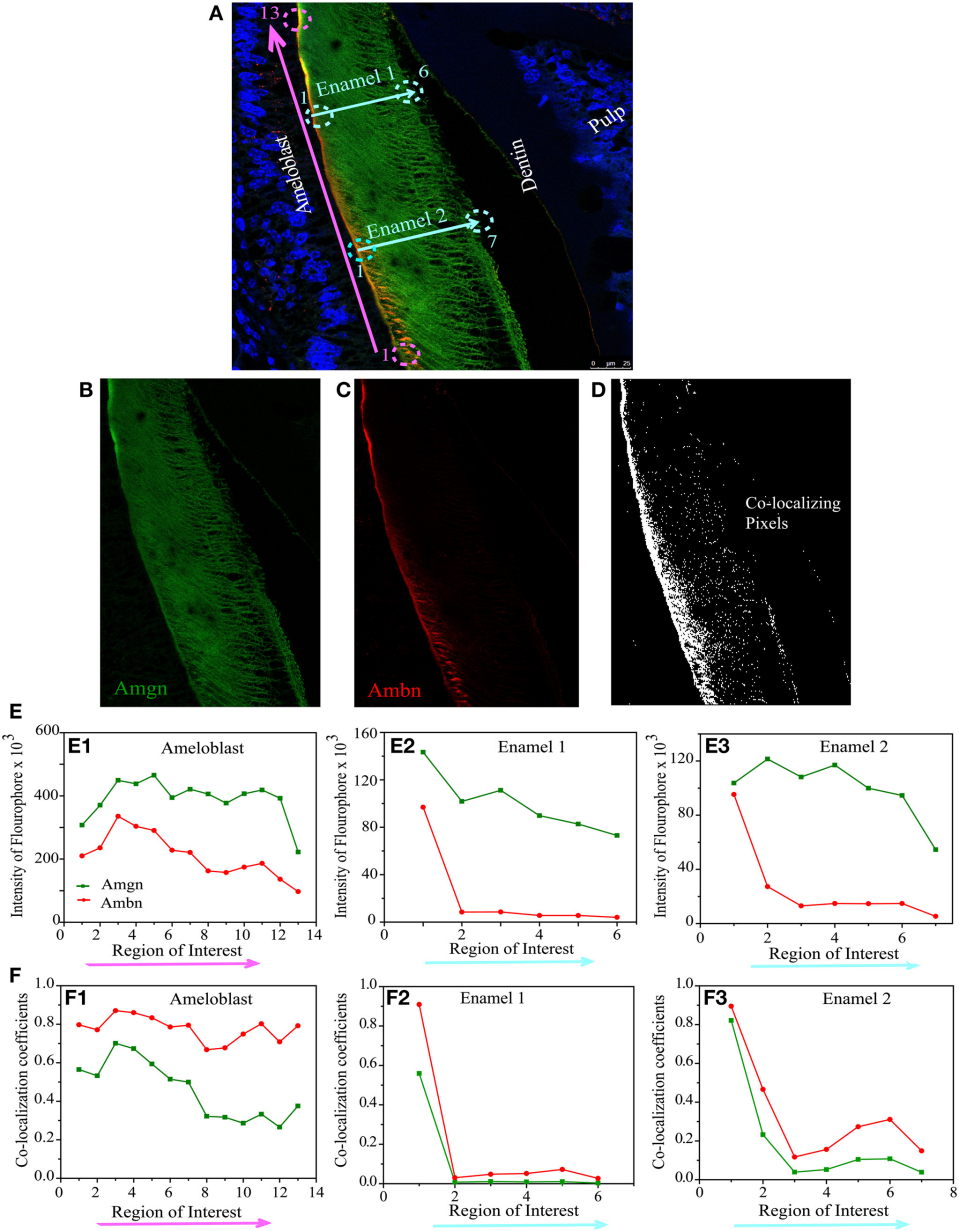
**Quantitative co-localization of ameloblastin and amelogenin in mouse molar enamel at P8. (A)** Confocal image with higher magnification taken from the sample in Figure 4C (see square), considered for QCA analysis along the enamel surface (pink arrow) and enamel thickness (cyan arrows). The individual channels detecting **(B)** only Amgn and **(C)** only Ambn signal. **(D)** Represents co-localization pattern after analysis of image **(A)** with threshold and background correction of each channel. **(E)** Represents the intensity sum of each fluophore at each region of interest. **(F)** Plot of Mander's co-localization coefficient with respect to region of interest as shown in **(D)**. Each ROI dimension (10 × 10 μm). Note that **(E1,F1)** correspond to pink arrow in **(A)** and **(E2,F2)** correspond to cyan arrow in **(A)** (Enamel 1), and **(E3,F3)** correspond to cyan arrows (Enamel 2).

To visualize the co-localization of ameloblastin and amelogenin and for further confirmation of distribution of these two proteins in enamel extracellular matrix we acquired confocal images of tissue sections (simultaneously immunostained with two fluorophore mixture) at P1 (Figure 4A), P5 (Figure 4B) and P8 (Figure 4C). In Figure 4 the regions which were yellow or orange in color represent the co-localization of both the protein. Next quantitative co-localization analysis (QCA) was performed using fluorescence software to quantify the percentage of co-localization of Ambn and Amgn at P1 and P8 (Figures 5, 6) using selected region of these images (Figure 4, for P5 data not shown) with higher magnification.

QCA was conducted at day 1 using a selected co-localization region from Figure 4A (white square), which presented in Figure 5A. We have divided this co-localization region (yellow colored) into 14 small regions of interest (ROIs) and the direction of analyzed region of interest in Figure 5 is indicated by pink arrow along the secretory face of the ameloblast (at the enamel surface) from the cervical loop to tip of the molar. Then Mander's co-localization coefficients were calculated to characterize the degree of overlap between two channels (Figure 5B, green for Amgn and Figure 5C, red for Ambn) in the image and to predict the contribution of each particular antigen to the areas with co-localization, which is the number of pixels that have a co-localizing signal (Figure 5D, represented by white dots). First the values for signal intensity sum of the green channel (Figure 5E, green line), red channel (Figure 5E, red line) and the co-localization intensity (data not shown) were generated at each ROI. Then the co-localization intensity was divided by the signal intensity to obtain co-localization coefficients (Figure 5F), which measured the proportion of overlap of each channel with the other. As can be seen from the co-localization coefficient graph, at the initial stage of enamel development (P1), Amgn and Ambn co-localized near the secretory face of ameloblasts with almost the same proportion and high coefficient values (Figure 5F).

On day 8, QCA was performed with a section of Figure 4C (square or inset), represented in Figure 6A. At P8 the enamel was more mature, we have calculated co-localization coefficients in two different configurations, one along the secretory face of the ameloblast from the cervical loop to tip of the molar (at the enamel surface indicated by pink arrow) and two along the thickness of the enamel layer from the enamel surface to dentino-enamel junction (indicated by cyan blue arrows as Enamel1 and Enamel2) (Figure 6A). The two individual channels of this confocal image were presented by Figure 6B (green channel for Amgn) and Figure 6C (red channel for Ambn) and the number of pixels that have a co-localizing signal in same image indicated by white dots in Figure 6D. We have calculated the total sum of green (Figure 6E, green line) and red (Figure 6E, red line) intensities along, (i) the ameloblast (Figure 6E1) and (ii) enamel thickness (Figures 6E2,E3). We observed that at day 8 the intensity difference between the two channels increased in both configurations (Figures 6E1–E3) relative to day 1 (Figure 5E). The co-localization coefficient plot at P8 indicated that Ambn co-localized with Amgn along the secretory face of ameloblasts, and their proportions of co-localization differed (Figure 6F1). There was a higher percentage of Ambn co-localizing with Amgn than Amgn co-localizing with Ambn. Along the enamel thickness moving award from the ameloblasts, no significant co-localization was observed (Figures 6F2,F3).

It appears that at an early stage both antigens (ameloblastin and amelogenin) are distributed in the same region with high spatial overlap, i.e., we can assume that they may be close enough to form co-assemblies, but with maturation ameloblastin has a high co-localization coefficient in comparison to amelogenin.

**Figure 7:**
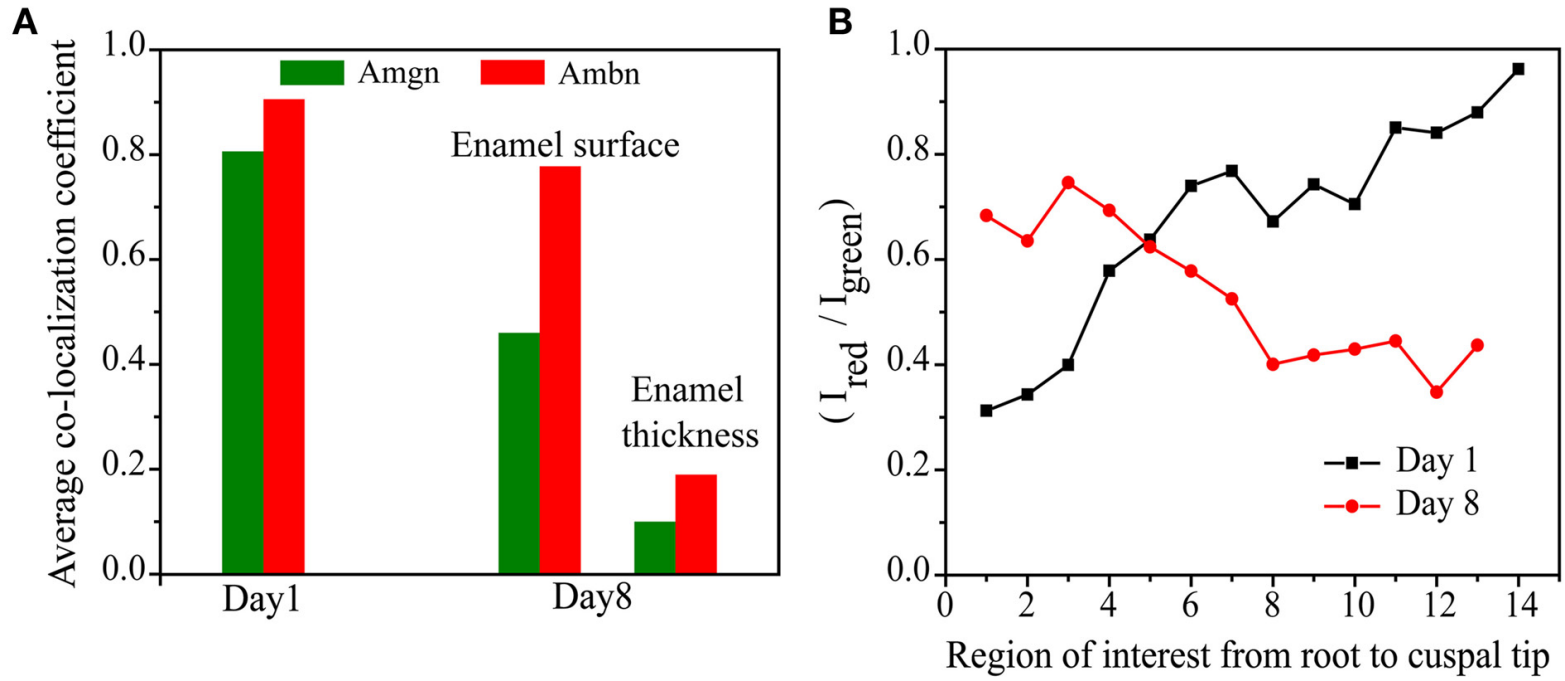
**Comparison of (A) the differences in the average co-localization coefficients of Amgn and Ambn at P1 and P8 and (B) intensity ratio of total sum of intensities of red to green channels of each region of interest along the enamel surface**.

This trend can be clearly seen when the average of the co-localization coefficients for each age is calculated and plotted (Figure 7A). We have calculated the ratio of the total intensity sum of Ambn-red channel (∑_*i*_*R*_*i*_) to the Amgn-green channel (∑_*i*_*G*_*i*_) of each region of interest (Figures 5E, 6E) along the enamel surface obtained from confocal images of day 1 and 8 mouse mandibular molars (Figure 7B). At an early developmental stage (day 1) we found that the ratio gradually reaches the value 1 when moving from the root to the tips of the tooth (Figure 7B, black line), which indicates that the proteins were secreted simultaneously and in similar proportions (calculated from QCA) from ameloblasts (Zalzal et al., 2008). With enamel maturation, at day 8 the intensity ratio decreases (Figure 7B, red line), indicating that at the maturation stage, ameloblastin and its proteolytic products (C-terminal large soluble polypeptides) (Lee et al., 1996; Uchida et al., 1997, 1998; Brookes et al., 2001) are gradually lost from the enamel surface.

Our co-localization studies suggested that amelogenin-ameloblastin complexes may be the functional entities at the early stage of enamel mineralization. In order to examine how amelogenin would affect ameloblastin's structure, we performed *in vitro* experiment, e.g., CD spectroscopy study with recombinant proteins.

### CD spectroscopic study of Ambn and Amgn interaction

**Figure 8:**
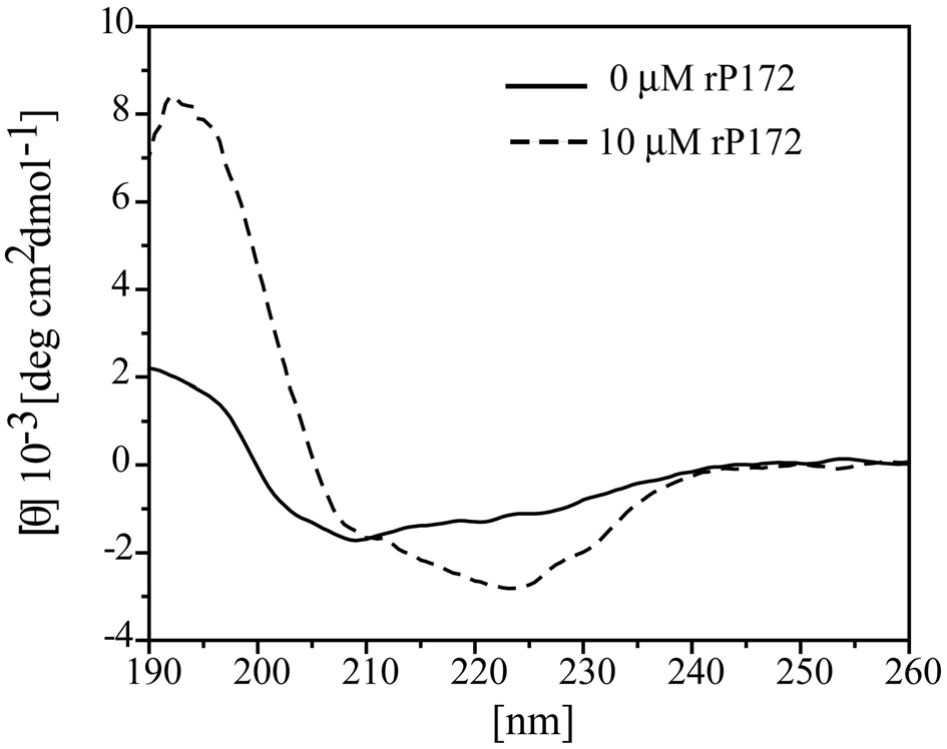
**Structural changes of ameloblastin (Ambn) in the presence of amelogenin (Amgn) provide evidence for their co-assembly**. Far-UV circular dichroism (CD) spectra of 10 μM rMAmbn only (solid line) and in the presence of 10 μM rP172 (dashed line).

CD spectra analysis of Ambn (10 μM) in the presence of equimolar Amgn revealed that Amgn induced changes in the secondary structure of Ambn (Figure 8). The CD spectrum of rMAmbn at 37 °C, with a negative minima around 208 nm and a small negative shoulder around 227 nm, is characteristic of an unordered structure (β II structure) (Figure 8, black solid line). Additionally, the spectra deconvolution shows rMAmbn contain intermixed alpha-helical (24%), beta-sheet (24%) and beta-turn (18%) regions with 34% unordered structure. The human recombinant full-length ameloblastin also exhibited β II like structural property at 37°C (Wald et al., 2011, 2013) and almost similar deconvolution pattern with less helical content and more beta character. With the addition of equimolar Amgn to Ambn, we observed an isoelliptic point appears at around 211 nm and a distinct change in CD bands. The intensity of the shoulder around 224 nm became more negative and at 192 nm a positive maxima was appeared. The spectral deconvolution exhibited the alpha-helical content increased to 51% and the beta-sheet, beta-turn and unordered forms decreased to 17, 12, and 14%, respectively. To avoid the interference of Amgn's structural changes on Ambn due to its self-association at high concentrations (>62.5 μM) (Lakshminarayanan et al., 2007), we used a very low concentration of Amgn (10 μM) and also we have subtracted its contribution from the CD spectra of equimolar mixture of both the proteins. The structural changes in Ambn at this low Amgn concentration thus clearly indicated that the proteins formed hetero-assemblies.

## Discussion

Amelogenin and ameloblastin, the major enamel matrix proteins, are important for well-orchestrated enamel biomineralization (Snead et al., 1985; Krebsbach et al., 1996). They have synergistic roles in enamel development, as suggested by recent study showing that Amel X^−/−^ /Ambn^−/−^ mice have additional enamel defects not observed in either Amel X^−/−^ or Ambn^−/−^ mice (Hatakeyama et al., 2009). The co-distribution of amelogenin and ameloblastin in the majority of the secretory granules in Tomes' processes during appositional growth of the enamel layer may reflect a form of functional association between these two distinct proteins (Zalzal et al., 2008). There is *in vitro* evidence of interactions between amelogenin (tyrosyl-binding motif) and ameloblastin (Ravindranath et al., 2004). The same group further produced some indications that during early tooth development the murine ameloblastin 37 kDa isoform interacts with amelogenin (Ravindranath et al., 2007). However, there still remains a gap in our knowledge of how these proteins are distributed in the extracellular enamel matrix, what the contribution pattern of each particular protein is to the areas with co-localization during the different stages of tooth development, and also what structural changes occur when amelogenin and ameloblastin co-assemble.

In order to give clear insight into the putative cooperative function of ameloblastin and amelogenin we took advantage of quantitative co-localization techniques and performed a systematic analysis of the localization of ameloblastin with respect to amelogenin in the mouse mandibular molar. We performed an additional *in vitro* study using circular dichroism (CD) spectroscopy to show the structural changes in ameloblastin when it co-assembles with amelogenin. We concentrated on confocal images from mandibular first molars from postnatal mice from one to eight days old (here the day of birth was termed postnatal day 0). Previous research into the expression of ameloblastin and amelogenin by *in situ* hybridization using DNA biotinylated probes (Torres-Quintana et al., 2005) has indicated that mRNA expression of ameloblastin and amelogenin in mouse molars is first observed in pre-ameloblasts at the tip of the cusp at postnatal day 2 for ameloblastin and day 3 for amelogenin (the day of birth was considered as postnatal day 1) and that expression of both proteins was seen until amelogenin expression terminated in maturation-stage ameloblasts at postnatal day 10. In our present study we used samples up to postnatal day 8 to ensure that both amelogenin and ameloblastin will be present (Figures 3A–D). The present study showed that secretion of amelogenin and ameloblastin starts at the cuspal slopes of molars, as seen on postnatal day 1 (Figures 3A, 4A, 5A). We observed that secretion continues until at least postnatal day 8 (Figures 3C, 4C, 6A). This is in agreement with the study of mRNA expression of ameloblastin (Torres-Quintana et al., 2005) but amelogenin was observed here earlier than the first detection of mRNA expression of amelogenin in the previous study. This difference could be explained by individual difference between mice and their strains.

Quantitative co-localization analysis is potentially a powerful tool for studying extracellular matrix protein interactions, as spatially related proteins have a high probability of interacting. Moreover, significantly more data can be generated while eliminating bias and errors of visual interpretation (Costes et al., 2004). In a recent report (Gallon et al., 2013) we have used confocal microscopy to successfully determine co-localization between amelogenin and enamelin in enamel extracellular matrix.

We utilized the Mander's co-localization coefficients M1 and M2 as these coefficients are not dependent upon the intensities of the signals and can compare signal intensities for different fluorophores when their signal intensities differ (Manders et al., 1993). These coefficients give a measure of the amount of fluorescence of the co-localizing objects of the image relative to the total fluorescence. By plotting the co-localization co-efficients M_green_ and M_red_ of amelogenin or ameloblastin against the regions of interest, patterns of co-localization were revealed and comparisons were made. The use of an antibody against ameloblastin may allow us to detect the full-length protein as well as its proteolytic products containing that epitopes. Such products may include the equivalent to the reported rat ameloblastin proteolytic products (Uchida et al., 1997, 1998; Brookes et al., 2001). While the pattern of proteolysis of porcine ameloblastin is well documented (Chun et al., 2010), information regarding the rodent proteolytic pattern is limited. Here the 62, 40, 33, and 29 kDa products were detected by Western blot analysis of protein extracted from 9-day-old mice (Figure 2, lane 3). We followed the migration pattern of proteolytic products from the ameloblastin C-terminal in the ECM at day 8. It is notable that signals for amelogenin and/or ameloblastin were not so prominent within the ameloblasts. Following their secretion and processing by MMP-20, epitopes on both amelogenin and ameloblastin can be more available for interaction with the antibody in the ECM.

As seen in Figure 7A, the proportion of co-localization between ameloblastin and amelogenin varies with the postnatal age of the mouse. At early stages, when enamel is initially developing, the proteins co-localize with each other to a large degree and in the same proportions (Figure 5F). Since we observe that the contribution of each antigen to the areas with co-localization is high, i.e., ameloblastin and amelogenin strongly overlap with each other at day 1, we interpret the data to suggest that at initial stages of enamel development they co-assemble. With enamel maturation this pattern changes. For a time, on day 8, the proportion of co-localizing ameloblastin remain greater than the proportion of co-localizing amelogenin, indicating there is now more “free” or non-co-localizing amelogenin than “free” ameloblastin. This also indicates that there is more amelogenin present in the extracellular matrix than ameloblastin at later stages, which is verified by plotting the intensity ratio with age (Figure 7B). These findings are in accordance with the published report that the C-terminal polypeptides of ameloblastin are successively cleaved into smaller peptides and lost from the extracellular matrix (Uchida et al., 1997, 1998) at maturation stage. Co-localization of amelogenin proteolytic products and ameloblastin N-terminal is possible and it is a subject of ongoing investigation (Mazumder et al., in preparation).

### Conflict of interest statement

The authors declare that the research was conducted in the absence of any commercial or financial relationships that could be construed as a potential conflict of interest.
